# An interpretable neural network for outcome prediction in traumatic brain injury

**DOI:** 10.1186/s12911-022-01953-z

**Published:** 2022-08-01

**Authors:** Cristian Minoccheri, Craig A. Williamson, Mark Hemmila, Kevin Ward, Erica B. Stein, Jonathan Gryak, Kayvan Najarian

**Affiliations:** 1 Department of Computational Medicine and Bioinformatics, University of Michigan, Ann Arbor, USA; 2 Department of Neurosurgery, University of Michigan, Ann Arbor, USA; 3 Max Harry Weil Institute for Critical Care Research and Innovation, University of Michigan, Ann Arbor, USA; 4 Department of Surgery, University of Michigan, Ann Arbor, USA; 5 Michigan Institute for Data Science (MIDAS), University of Michigan, Ann Arbor, USA; 6 Department of Emergency Medicine, University of Michigan, Ann Arbor, USA; 7 Department of Electrical Engineering and Computer Science, University of Michigan, Ann Arbor, USA; 8 Department of Radiology, University of Michigan, Ann Arbor, USA

**Keywords:** Traumatic brain injury, Outcome prediction, Interpretable machine learning, Neural networks, Clinical decision support systems

## Abstract

**Background:**

Traumatic Brain Injury (TBI) is a common condition with potentially severe long-term complications, the prediction of which remains challenging. Machine learning (ML) methods have been used previously to help physicians predict long-term outcomes of TBI so that appropriate treatment plans can be adopted. However, many ML techniques are “black box”: it is difficult for humans to understand the decisions made by the model, with post-hoc explanations only identifying isolated relevant factors rather than combinations of factors. Moreover, such models often rely on many variables, some of which might not be available at the time of hospitalization.

**Methods:**

In this study, we apply an interpretable neural network model based on tropical geometry to predict unfavorable outcomes at six months from hospitalization in TBI patients, based on information available at the time of admission.

**Results:**

The proposed method is compared to established machine learning methods—XGBoost, Random Forest, and SVM—achieving comparable performance in terms of area under the receiver operating characteristic curve (AUC)—0.799 for the proposed method vs. 0.810 for the best black box model. Moreover, the proposed method allows for the extraction of simple, human-understandable rules that explain the model’s predictions and can be used as general guidelines by clinicians to inform treatment decisions.

**Conclusions:**

The classification results for the proposed model are comparable with those of traditional ML methods. However, our model is interpretable, and it allows the extraction of intelligible rules. These rules can be used to determine relevant factors in assessing TBI outcomes and can be used in situations when not all necessary factors are known to inform the full model’s decision.

## Background

Traumatic Brain Injury (TBI) is a very common medical problem with the potential for severe harm [6, 18]. In 2017, TBIs were identified in 25% of all injury-related deaths in the United States. Every year, well over one million Americans sustain a form of TBI, resulting in over 200,000 hospitalizations and leaving survivors with disabilities that require years of rehabilitation at significant healthcare cost. Despite the magnitude of this problem, few effective treatments are available. For decades there have been efforts towards developing diagnostic and treatment coupled pathways [22], followed by those considering additional risk factors. Numerous studies have found correlations between variables known at the time of hospital admission and mortality; however, it has proven difficult to find general and comprehensive guidelines for assessment and decision making [16]. A serious challenge in severe TBI consists of acutely determining whether a patient should undergo continued life-sustaining treatment, since early withdrawal of care commonly results in death [8]. Sometimes “self-fulfilling prophecies” [4] are caused by early withdrawal of treatment when a prognostic factor is found, whose relevance is then reinforced by the outcome.

In recent years, several studies have adopted machine learning methods to predict mortality in patients admitted with TBI [1, 2, 10, 14, 17, 19]. However, these studies focused on in-hospital or early mortality. Fewer studies have investigated longer term outcomes that incorporate functionality in addition to mortality [3, 7, 11, 20, 23, 24]. Furthermore, it has been observed how this kind of problem is especially challenging even for machine learning (ML) methods, which often perform no better than linear regression [9]. Another fundamental issue of many ML models in healthcare applications is their “black box” nature, i.e., their lack of interpretability. This has greatly hindered their adoption since clinicians need to be able to understand how such models reach conclusions in order to validate the results and/or integrate them into their decision making. Additionally, a known problem of ML approaches to TBI assessment is that they usually rely on a large number of variables that might not be available at the time of hospitalization [13]. Our previous work [5] has addressed some of these issues by predicting the recovery outcome at six months from hospitalization and by building a framework for an intelligible TBI prognostic model. However, this methodology required expert validation, and could only identify relevant prognostic factors, rather than rules (combinations of multiple factors).

A method to make models intelligible is the use of fuzzy logic. Fuzzy logic and fuzzy inference models [21, 28] are established methods for both integrating humanly understandable rules into ML models and extracting understandable rules. A key idea is the use of membership functions to measure the extent to which a crisp value *x* belongs to a given fuzzy concept. For example, we might have a membership function *l* (typically a triangular function) for the fuzzy concept of “low”, and the value *l*(*x*) representing the degree to which *x* can be considered to be “low”. This approach can be combined with neural networks in the form of adaptive network-based fuzzy inference systems [12]. In our previous work [26], we used a genetic algorithm to train a fuzzy neural network to recommend treatments for advanced heart failure patients. That work was generalized and refined by introducing tropical geometry into the model [27] to make it more flexible by parametrizing the aggregation operations and membership functions.

In this study, we apply the intelligible neural network model based on fuzzy logic and tropical geometry that first appeared in [27] to predict the recovery from TBI at six months from hospitalization. While our previous paper was focused on the algorithmical development of the method, in this work we focus on its clinical application to TBI assessment, as well as on ways of enhancing interpretability and controlling the number concepts within each rule to be extracted. The model allows us to extract rules that can be understood by humans, making it highly interpretable since its decision process is transparent. Additionally, each of these rules only involves a few factors, so that they can be used individually if some of the variables are not available. The model is extensively tested on different sets of variables and using different loss terms to further investigate its capabilities. The classification results of the various regularized versions of the proposed model are comparable to each other and to the other ML algorithms we considered. Moreover, the proposed model allows us to interpret the classification results and to extract general, humanly understandable rules while retaining good classification performance.

## Methods

The Tropical geometry-based Fuzzy Neural Network (TFNN) used in this work was introduced in [27]. Tropical geometry can be thought of as a piecewise linear version of algebraic geometry, where usual addition and multiplication are replaced by $\max$ (or $\min$) and by addition, respectively. Connections between tropical geometry and neural networks have been partially explored, as in [29], but not widely so. The proposed model uses the tropical framework in a new way, by interpolating between a traditional, smooth neural network and a fuzzy one. Fuzzy inference models replace crisp membership functions with fuzzy ones, such as triangular or trapezoidal membership. This allows one to replace the crisp value of a continuous variable with the fuzzy concepts of “low”, “medium”, and “high”, by defining to what extent the crisp value belongs to each of these concepts. One key advantage is the ability to extract humanly understandable knowledge from the data, in the form of “if-then” rules that can prove valuable in the decision making process. Furthermore, the fuzzy framework enables domain knowledge in the form of rules already known to experts to be incorporated into the learning process; this way, we can improve the training of the model both in terms of performance and by reducing the amount of training data required.

However, it is not obvious a priori which membership function will be best suited for a given task, as well as which aggregation operations (minimum and product, or maximum and addition). Tropical geometry allows us to interpolate between fully piecewise linear operations and smooth ones, as well as between aggregation operations. This provides the model with additional flexibility, allowing it to learn the optimal membership functions and aggregation operations. Additionally, the optimization process works with smooth functions (updated during training to be closer to piecewise linear ones), allowing us to use a gradient descent algorithm which couldn’t previously be used within fuzzy frameworks.

**Fig. 1:**
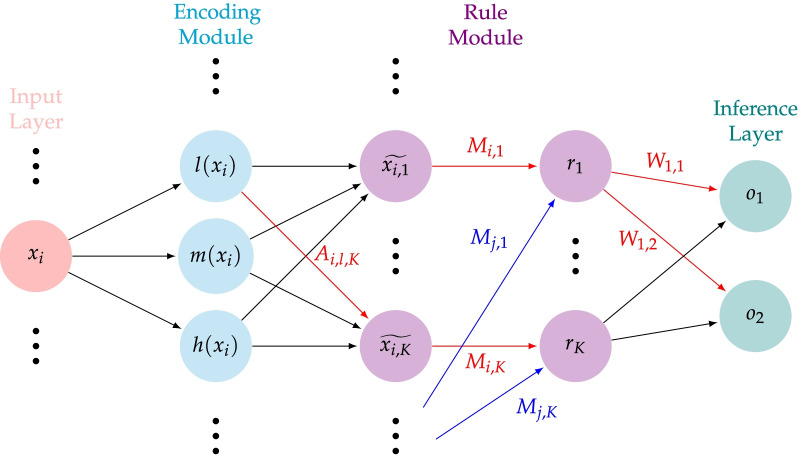
A schematic of the tropical geometry-based neural network introduced in [27]

We refer the reader to [27] for a full description of the model. We report in Fig. 1 a schematic diagram of the layers, and briefly describe its components.

In the encoding module, a continuous variable $x_i$ is assigned three values in [0,1] representing the membership value to the concepts of “low” ($l(x_i)$), “medium” ($m(x_i)$), and “high” ($h(x_i)$). The number of concepts can be varied (e.g., 2, 4) to suit the application. Unlike in traditional fuzzy theory, membership functions depend on a trainable parameter $\epsilon$ which determines their smoothness.

In the rule module, a total of *K* rules $r_1,\ldots ,r_K$ are constructed. The weights of the first layer of the rule module constitute the attention matrix *A*, obtained by concatenating submatrices $A_{i,:,:}$, one for each input variables. A higher value corresponds to a higher contribution of the associated concept to the associated rule. The weights of the second layer of the rule module—whose nodes $r_1,\ldots ,r_K$ correspond to the rules to be extracted—constitute the connection matrix *M*. An entry $M_{i,k}$ represents the importance of the *i*th input variable to the construction of the *k*th rule. The outputs $r_1,\ldots ,r_K$ are computed via a parametrized norm (dependent on the trainable parameter $\epsilon$) interpolating between the operations of product and minimum. Weights of both the attention and connection matrix are learned and constrained to [0,1] via a hyperbolic tangent activation function.

Finally, the inference module consists of a fully connected layer with nodes corresponding to the classification categories $o_1,\ldots ,o_C$ (in our study, the number of classes is $C=2$). The positive weights $W_{k,c}$ of this layer are learned and correspond to the contribution of the *k*th rule to the *c*th class. Each output $o_c$ is computed via a parametrized conorm (dependent on the trainable parameter $\epsilon$) interpolating between the operations of sum and maximum.

Once the network is trained, rules can be extracted from the weights. For the *k*th rule, a contribution matrix $S_{:,:,k}$ can be constructed, with entries computed from the attention and connection matrices as $S_{i,d,k}=A_{i,d,k}M_{i,k}$. Here *i* represents the index of the input variable $x_i$, $d=l,m,h$ represents each of the concepts of “low” (*l*) “medium” (*m*) and “high” (*h*), and $k=1, \ldots , K$ represents the *k*th rule. The value $S_{i,d,k}$ represents the contribution of the *d*th concept of the *i*th input variable to the *k*th rule. The value $W_{k,c}$ represents the importance of the *k*th rule in determining whether the input data belongs to the *c*th class. Therefore, for each *k*, the *k*th rule is fully captured by the matrix $S_{:,:,k}$ and the value $W_{k,:}$. In our study, $C=2$ and when discussing rules we will consider those contributing to the positive class. To exemplify this, assume that the trained model identified rule $r_1$ as “$x_i$ low” AND “$x_j$ high”, and as relevant for the first class. That means that in the model $W_{1,1}$ would be high (meaning that rule $r_1$ is relevant for the class $o_1$) and that $S_{i,l,1}$ (the importance of the concept of $x_i$ being low in building the first rule) and $S_{j,h,1}$ (the importance of the concept of $x_j$ being high in building the first rule) would be high.

The network is trained with the Adam optimizer via backpropagation. The total loss is computed as $loss_{ce}+\lambda _1 loss_{sparse}+\lambda _2 loss_{corr}$, where $loss_{ce}$ is a standard cross-entropy loss term, $loss_{sparse}$ is a sparsity term to penalize rules with too many variables, and $loss_{corr}$ is a correlation term to penalize the extraction of redundant rules. Specifically, we consider two forms of $loss_{sparse}$:

$$loss_{sparse}=loss_{\ell _i}=\Vert vec(A)\Vert _i+\Vert vec(M)\Vert _i$$

for $i=0,1$ (where $\Vert vec(A)\Vert _i$ and $\Vert vec(M)\Vert _i$ are the $\ell _i$-norm of the attention matrix *A* and the connection matrix *M* respectively). Finally, the correlation loss is computed as

$$loss_{corr}=\sum _{i=1}^{H-1} \sum _{j=i+1}^H vec(S_{:,:,i})vec(S_{:,:,j}),$$

where *H* is the number of input variables.

## Dataset and methodology

The ProTECT III dataset (Progesterone for Traumatic Brain Injury Experimental Clinical Treatment) was collected for a research study with the goal of testing whether progesterone treatment for TBI is safe and/or effective [25]. All methods were performed in accordance with the relevant guidelines and regulations. The dataset consists of 882 patients with electronic health record (EHR) data collected at the time of hospitalization; among these, we excluded those with non-survivable injuries, and only considered the remaining 833. Long-term recovery from TBI is assessed via the Glasgow Outcome Scale Extended (GOSE), a global scale to evaluate recovery at 6 months from insult. GOSE is the most commonly used measure of TBI outcome assessment and its validity has been corroborated by prior studies [15]. Among the 833 patients, 350 have GOSE 1-4 and constitute the class of patients with negative recovery outcome (from severe disability to death), and 483 have GOSE 5-8 and constitute the class of patients with positive recovery outcome (from moderate disability to good recovery). After excluding features such as race and cause of injury (with the goal of only including strictly medical features), a total of 58 features per patient remained. The proposed model was trained using three different sets of features: all 58 features, 18 robust features selected using SHapley Additive exPlanations (SHAP) in [5], and the best 18 features computed from the Minimum Redundancy Maximum Relevance (MRMR) algorithm. With respect to MRMR, there are 13 features with scores above 0.01 and 18 features with scores above 0.007. We selected the 18 highest-scoring features—described in Table 1—for better comparison with the features in [5]; 12 of the 18 features selected via MRMR overlap with the 18 features selected via SHAP.

**Table 1:** Features with the highest MRMR scores. Variables sourced from radiology reports are suffixed with *rad.*

Variable	MRMR score
Subarachnoid hemorrhage (#)—rad.	0.0679
Intraparenchymal hematoma—rad.	0.0631
DAI finding—rad.	0.0683
Third ventricle compression—rad.	0.0355
Best motor response—baseline	0.0275
Age—demographics	0.0261
Pupil response—baseline	0.0258
Intra-ventricular hemorrhage—rad.	0.0198
Skull fracture: basilar—rad.	0.0147
Best eye opening—baseline	0.0128
Brain contusion (#)—rad.	0.0121
Herniation: transtentorial—rad.	0.0106
Subdural hematoma—rad.	0.0101
Intraparenchymal hematoma (max width)—rad.	0.0087
Abnormal finding—rad.	0.0085
Best verbal response—baseline	0.0078
Herniation: upward—rad.	0.0077
Herniation: uncal—rad.	0.0072

The proposed model along with established ML models (Random Forest, SVM, XGBoost) was tested on the ProTECT III dataset; the reported results are the average scores of 10-fold cross-validation.

## Results

Tables 2, 3 and 4 contain relevant performance metrics of the proposed model (TFNN) in comparison to XGBoost (XGB), Random Forest (RF), and Support Vector Machine (SVM), for each set of features we considered. In this set of experiments, TFNN is run with no sparsity loss term (i.e., with total loss computed as $loss_{ce}+\lambda loss_{corr}$) and with a total number of rules optimized among 20, 25, and 30. The performance of the proposed model is always very close to that of the best performing traditional machine learning method, with the crucial difference that TFNN is interpretable and provides intelligible rules. The worse performance using all 58 features is to be expected for a neural network, given the small size of the dataset compared to the number of features used.

Table 5 compares different versions of the proposed model on each set of features considered. “TFNN” refers to the model run with no sparsity loss term and with a total number of rules optimized among 20, 25, and 30, as before; “fewer rules” refers to the model run with no sparsity loss term but a smaller total number of rules, optimized among 5, 10, and 15; “$\ell _1$-sparsity” and “$\ell _0$-sparsity” refer to the model run with a sparsity loss term. We observed that a larger number of rules provides better results, as well as an $\ell _0$-sparsity term rather than an $\ell _1$-sparsity term. The main effect in imposing sparsity is that of extracting simpler rules with fewer variables.

**Table 2:** Mean (standard deviation) of performance metrics using 18 features selected in [5] via SHAP

Method	Accuracy	Recall	Precision	F1	AUC
TFNN	0.719 (0.040)	**0.657** (0.094)	0.671 (0.054)	**0.614** (0.057)	0.794 (0.039)
XGB	0.693 (0.028)	0.591 (0.075)	0.646 (0.033)	0.569 (0.048)	0.743 (0.039)
RF	**0.744** (0.035)	0.579 (0.055)	**0.754** (0.057)	0.608 (0.049)	**0.802** (0.036)
SVM	0.728 (0.019)	0.551 (0.106)	0.745 (0.056)	0.579 (0.059)	0.795 (0.048)

Values in bold are the highest for a given metric across different methods

**Table 3:** Mean (standard deviation) of performance metrics using the best 18 features selected by MRMR

Method	Accuracy	Recall	Precision	F1	AUC
TFNN	0.719 (0.033)	**0.617** (0.074)	0.683 (0.046)	0.600 (0.050)	0.793 (0.039)
XGB	0.675 (0.027)	0.584 (0.053)	0.619 (0.033)	0.554 (0.039)	0.716 (0.034)
RF	**0.740** (0.031)	0.581 (0.053)	**0.744** (0.051)	**0.606** (0.044)	**0.800** (0.037)
SVM	0.731 (0.035)	0.590 (0.055)	0.719 (0.053)	0.601 (0.047)	**0.800** (0.040)

Values in bold are the highest for a given metric across different methods

**Table 4:** Mean (standard deviation) of performance metrics using all 58 features

Method	Accuracy	Recall	Precision	F1	AUC
TFNN	0.702 (0.026)	0.551(0.050)	0.684 (0.038)	0.564 (0.039)	0.786 (0.027)
XGB	0.697 (0.019)	**0.624**(0.041)	0.647 (0.026)	0.588 (0.026)	0.762 (0.016)
RF	0.735 (0.021)	0.574 (0.054)	**0.741** (0.027)	0.599 (0.038)	**0.810** (0.018)
SVM	**0.740** (0.018)	0.615 (0.048)	0.731 (0.037)	**0.620** (0.028)	0.808 (0.024)

Values in bold are the highest for a given metric across different methods

**Table 5:** Mean AUCs of several variants of the proposed model

Method	SHAP features	MRMR features	All 58 features
TFNN	0.794 (0.039)	0.793 (0.039)	**0.786** (0.027)
$\ell _1$-sparsity	0.788 (0.033)	0.794 (0.041)	0.765 (0.022)
$\ell _0$-sparsity	**0.799** (0.035)	**0.797** (0.041)	0.784 (0.030)
fewer rules	0.784 (0.037)	0.784 (0.042)	0.775 (0.022)

Values in bold are the highest for a given metric across different methods

### Extracted rules

**Fig. 2:**
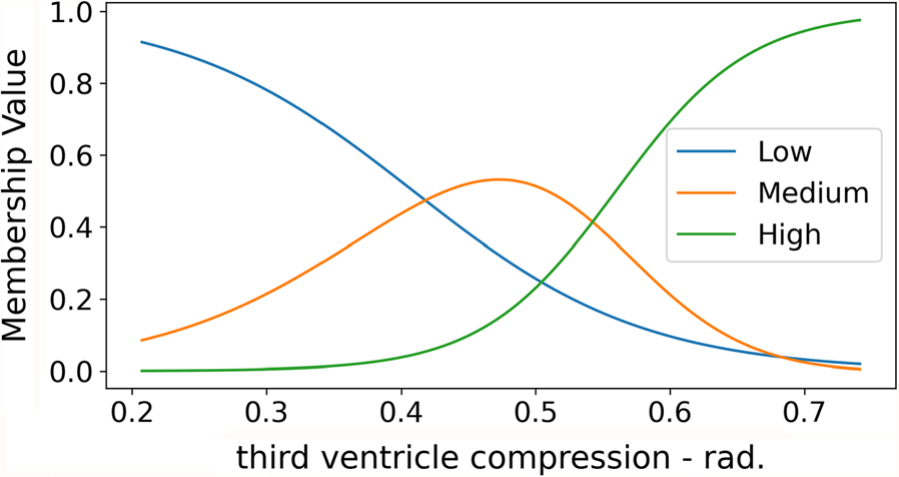
Membership functions for the concepts of low, medium, and high of the variable “third ventricle compression—rad.”, extracted from the model trained on SHAP features with $\ell _0$-sparsity

Presented below are the most relevant rules selected by the TFNN model on two sets of features: SHAP features and MRMR features. The rules are extracted from the model trained on the first 9 of the 10 folds. The rules correspond to the nodes $r_1, \ldots , r_K$ of the rule module; for the sake of interpretability and ease of visualizations, rules with high correlation and concepts with low contribution to a rule are removed. The best performing model from Table 5 is the $\ell _0$-sparsity model using SHAP features; we will compare it to the a model trained on the same features but without a sparsity term. We then describe rules extracted from MRMR features without sparsity terms. For each group of features, the presented rules are those the model deemed most important, i.e., the rules with the highest weights. Another important layer of interpretability consists of having a range for each of the concepts of low, medium, and high, learned by the network. For example, Fig. 2 shows the final membership functions learned by the model with the $\ell _0$-sparsity term on SHAP features for the variable “third ventricle compression—rad.”. Each input value can be low, medium, or high to some degree, and the network additionally determines the range for each concept. For example, the concept of high for the “third ventricle compression—rad.” variable is computed as having values higher than 0.59.

In the case of SHAP features with the $\ell _0$-sparsity term, a total of 30 rules are extracted (equivalently, the rule module has 30 nodes). The rules refer to the class with GOSE 1-4 that corresponds to poor recovery outcome. We report here the three most important—and not highly correlated—rules, i.e., those with highest weights. For this set of rules, next to each concept is the numerical value that defines it according to the model. We denote with the concept “not low” the union of the concepts “medium” and “high”.

1. **IF** *intra-ventricular hemorrhage—rad. high* ($>0.5$) **AND** *best eye opening—baseline low* ($<1.6$) **AND** *best verbal response—baseline low* ($<2.1$) **AND** *best motor response—baseline low* ($<4.1$) **AND** *DAI finding (#)—rad. high* ($>0.9$);
2. **IF** *subdural hematoma (#)—rad. not low* ($>0.8$) **AND** *age high* ($>48$);
3. **IF** *third ventricle compression—rad. high *($>0.4$) **AND** *herniation: transtentorial—rad. high* ($>0.49$).

For this model, it wasn’t necessary to remove concepts with low contribution to the rules because of the addition of a sparsity term in the training of the model, which forced the rules to depend on fewer concepts.

To verify that the extracted rules are meaningful, we can consider the patients in the dataset that satisfy them. There are a total of 59 patients satisfying Rule 1. Though this number is not high, it is reasonable as the rule includes many concepts and is therefore somewhat restrictive. Of these, 44 have GOSE less than 5. If the next most important rule, Rule 2, is included, there are only 5 patients satisfying both rules, all of whom have GOSE less than 5. If we consider Rule 2, a total of 166 patients satisfy it. This is a larger set than for Rule 1, which is to be expected, as Rule 2 only involves two concepts. However, 121 of these patients have GOSE less than 5, meaning that even a rule with only two concepts can be very significant.

In the next two cases we consider, we didn’t apply a sparsity term; as a consequence the rules depend on more concepts. In order to make the rules more intelligible, concepts with low contribution to a rule are removed.

The main rules (for the class with GOSE 1-4) extracted from the 18 features selected from SHAP values without a sparsity term are the following:

1. **IF** *subarachnoid hemorrhage (#)—rad. high* **AND** *intra-ventricular hemorrhage—rad. high* **AND** *best verbal response—baseline low* **AND** *best motor response—baseline low*;
2. **IF** *intra-ventricular hemorrhage—rad. high* **AND** *best verbal response—baseline low***AND** *Hgb lab low*;
3. **IF** *intraparenchymal hematoma—rad. high* **AND** *best verbal response—baseline low*.

There are a total of 48 patients in the dataset that satisfy this rule, 44 of which have GOSE less than 5. Of the 4 that do not satisfy the rule, the patient with the highest GOSE has a value of 7, which would be the worst misclassification. However, this patient would not satisfy the next rule, Rule 1, since their Hgb lab value is 14, whereas the concept of low corresponds to less than 13.2.

**Fig. 3:**
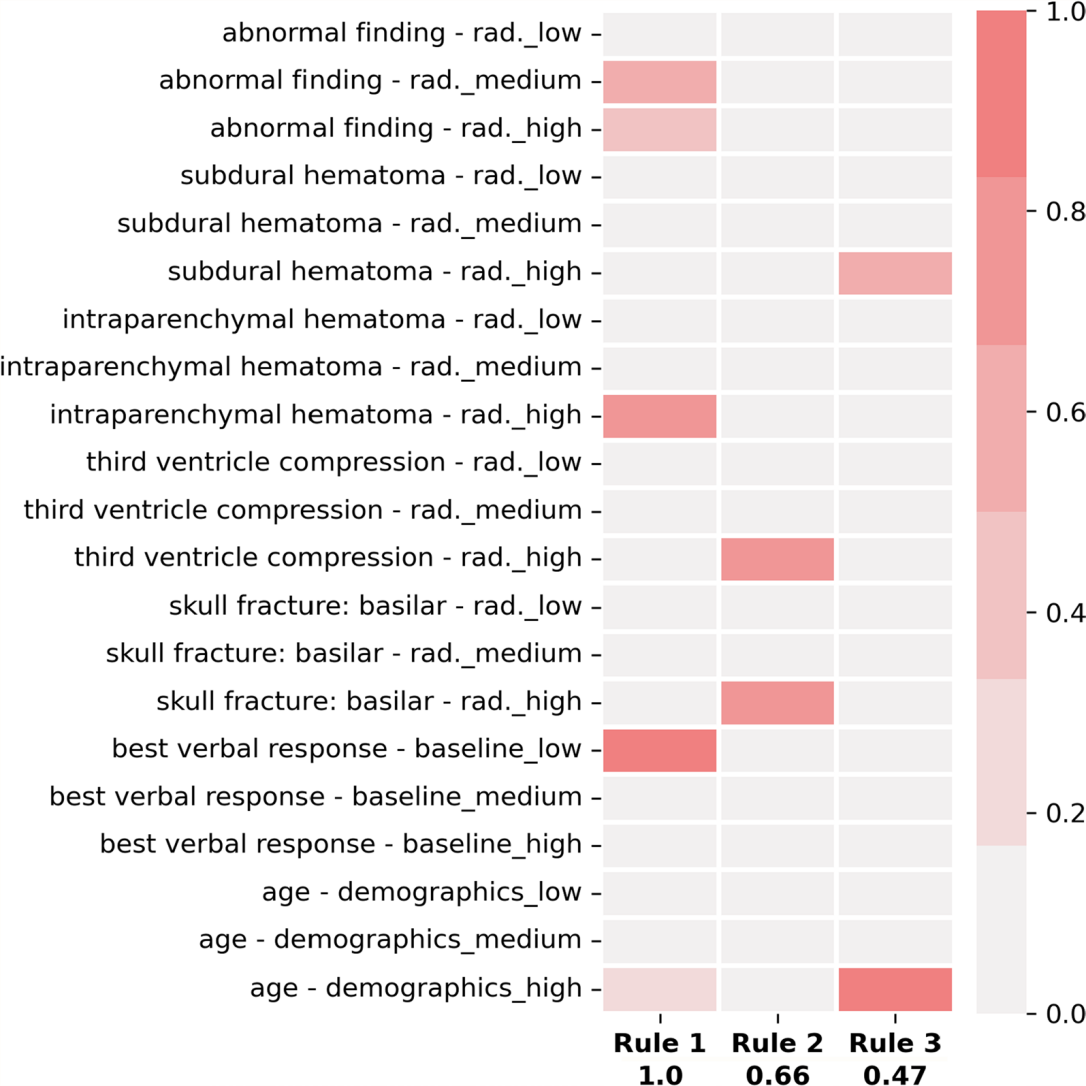
Rules extracted from MRMR features without a sparsity term

For completeness, we can consider another set of rules (for to the class with GOSE 1-4) without a sparsity term, namely the main rules extracted from the set of MRMR features (also depicted in Fig. 3):

1. **IF** *abnormal finding—rad. not low* **AND** *intraparenchymal hematoma—rad. high* **AND** *best verbal response—baseline low* **AND** *age—demographics high*.;
2. **IF** *third ventricle compression—rad. high* **AND** *skull fracture: basilar—rad. high*;
3. **IF** *subdural hematoma—rad. high* **AND** *age—demographics high*.

Except for “abnormal finding—rad.” and “skull fracture: basilar - rad.”, these features also belong to the set of SHAP features, and are therefore consistently recognized as meaningful by the model. Not imposing a sparsity term allows the first rule to be somewhat complex even after removing concepts with low relevance. However, the second and third most relevant rules of this model, shown in Fig. 3, depend only on two factors.

A similar analysis to the one conducted previously shows that within the entire dataset, 17 patients satisfy Rule 1, and among these only one patient has GOSE greater than 4 (namely, 7). However, this patient would fail Rule 2 since both variables “third ventricle compression” and “skull fracture” have a value of 0, and therefore neither is high.

## Discussion

The classification results for the proposed model are comparable with those of XGBoost, Random Forest, and SVM. In terms of AUC, the best performing model across all considered sets of features is Random Forest, with the best result achieved on the set of all 58 features. However, when considering values one standard deviation from the average AUC, the intervals of the proposed model and Random Forest overlap, meaning that the difference in performance is not substantial. The worst AUC performance of our model is on the set of all 58 features, which is to be expected from a NN based model given the reduced size of the dataset. Reducing the number of features improves the classification results. However, reducing the number of rules (equivalently, the nodes in the last hidden layer) consistently amounts to a worse performance. Introducing sparsity terms doesn’t affect AUC scores in a significant way: the change is negligible, and can lead to higher or lower average AUC depending on the set of features we considered. However, on the given dataset $\ell _0$-sparsity is preferred over $\ell _1$-sparsity. While the change in AUC is large and consistent enough to make a definitive conclusion, the main effect of imposing sparsity terms is on the set of rules: sparsity leads to “simpler” rules with fewer variables. Therefore, the choice of whether to use sparsity should be mainly guided by the type of rules that would most useful for a given application. The general rules extracted from the model have been analyzed by clinicians and make clinical sense. The rules are human-understandable, making use of concepts such as “low” and “high”, with the numerical thresholds defining these concepts being readily extractable if needed. From the results, one can observe that the rules are also meaningful in the sense that they capture a majority of the population with low GOSE score.

There are some limitations of our study, mainly related to the utilized dataset. Despite ProTECT III being one of the largest available dataset for TBI, the size doesn’t allow for the presented results to be considered definitive, as they should be tested on larger and different datasets as well. Additionally, the patients in our study lean towards severe outcomes of TBI, and a study more inclusive of milder TBI outcomes would be beneficial. Another limitation is the classification by means exclusively of the GOSE score: despite this being a crucial measure of TBI outcome, it does not capture all potentially relevant post-traumatic conditions, and it is not sufficient to paint an exhaustive picture of a patient’s recovery. Finally, the data utilized for each patient was generally the most proximal data available at the time of hospitalization. The severity of the injury as well as the variables of interest can change, and a more comprehensive model would take that into account as well. Future studies should consider larger datasets, multiple measures to assess TBI outcome, and examine the use of the algorithm for updated prognostics.

## Conclusions

TBI is a common and challenging health care issue which has proved difficult to impact by ML methods which are typically “black box” and incapable of providing explanations for their decision process. The classification results for the proposed model are comparable with those of traditional ML methods. However, unlike other ML algorithms, our model is interpretable, since it allows for an explanation as to why a patient was classified in a certain way by looking at rules the neural network determined to be relevant. These rules are intelligible; as such clinicians can assess whether they are sensible and therefore whether the result is reasonable resulting in clinical support tool with credibility. Additionally, these rules can be used to determine relevant factors in assessing TBI outcomes as well as serve as prognostication tools. Finally, by dividing the decision process in simpler components, our model allows the use of single rules or small groups of rules in situations when not all necessary factors are known to inform the full model’s decision.

## Data Availability

The data that support the findings of this study are available from Progesterone for Traumatic Brain Injury Experimental Clinical Treatment (ProTECT) III Trial’s Principal Investigator David Wright but restrictions apply to the availability of these data, which were used under license for the current study, and so are not publicly available. Data are however available from Jonathan Gryak upon request and with permission of Progesterone for Traumatic Brain Injury Experimental Clinical Treatment (ProTECT) III Trial’s Principal Investigator David Wright.
